# Neuroimaging of Traumatic Brain Injury

**DOI:** 10.3390/medsci7010002

**Published:** 2018-12-20

**Authors:** David B. Douglas, Tae Ro, Thomas Toffoli, Bennet Krawchuk, Jonathan Muldermans, James Gullo, Adam Dulberger, Ariana E. Anderson, Pamela K. Douglas, Max Wintermark

**Affiliations:** 1 Department of Neuroradiology, Stanford University, Palo Alto, CA 94301, USA; ddouglas@stanford.edu or david.b.douglas.mil@mail.mil; 2 Department of Radiology, David Grant Medical Center, Travis AFB, CA 94535, USA; tae.h.ro.mil@mail.mil (T.R.); thomas.j.toffoli.mil@mail.mil (T.T.); bennet.d.krawchuk.mil@mail.mil (B.K.); jonathan.l.muldermans.mil@mail.mil (J.M.); james.j.gullo4.mil@mail.mil (J.G.); adam.r.dulberger.mil@mail.mil (A.D.); 3 Department of Psychiatry and Biobehavioral Sciences, UCLA, Los Angeles, CA 90095, USA; ariana82@ucla.edu (A.E.A.); pamelita@ucla.edu or pdouglas@ist.ucf.edu (P.K.D.); 4 Institute for Simulation and Training, University of Central Florida, Orlando, FL 32816, USA

**Keywords:** concussion, traumatic brain injury, TBI, diffusion tensor imaging, perfusion imaging

## Abstract

The purpose of this article is to review conventional and advanced neuroimaging techniques performed in the setting of traumatic brain injury (TBI). The primary goal for the treatment of patients with suspected TBI is to prevent secondary injury. In the setting of a moderate to severe TBI, the most appropriate initial neuroimaging examination is a noncontrast head computed tomography (CT), which can reveal life-threatening injuries and direct emergent neurosurgical intervention. We will focus much of the article on advanced neuroimaging techniques including perfusion imaging and diffusion tensor imaging and discuss their potentials and challenges. We believe that advanced neuroimaging techniques may improve the accuracy of diagnosis of TBI and improve management of TBI.

## 1. Introduction

The Centers for Disease Control and Prevention (CDC) defines a traumatic brain injury (TBI) as a “bump, blow or jolt to the head, or penetrating head injury, that results in disruption of the normal function of the brain” [1]. Traumatic brain injury is major cause of morbidity and mortality worldwide and results from a wide range of injuries. In the civilian populations, common causes include falls, motor vehicle accidents, assault, and sports-related injuries. In the military population, common causes include blast injuries and penetrating trauma [2,3,4,5]. 

The purpose of this article is to provide a review conventional diagnostic neuroimaging techniques and advanced neuroimaging techniques including perfusion imaging and diffusion tensor imaging (DTI) as they apply to TBI. We will also discuss future research directions.

### 1.1. Neuroanatomy

The human brain is comprised of 100 billion neurons that communicate with each other through a complex network [6]. Neuron cell bodies, which form the cerebral cortex, communicate over short distances via a complex, branching network of dendrites and over long distances via axons. Axons travel in clusters called tracts to remote regions of the brain. Axons are wrapped in myelin, which serves to insulate the traveling electrical signal and connect functionally specialized yet segregated regions of the brain. As any neurosurgeon would tell you, this extremely complex architecture of the human brain is soft to the touch. And, it is vulnerable to direct injury from trauma—acceleration, deceleration, shearing forces, penetrating injury—as well as secondary injury from mass effect or ischemia etc.

### 1.2. Severity of Traumatic Brain Injury

In 1974, a landmark paper titled “Assessment of Coma and Impaired Consciousness: A Practical Scale” was written by Graham Teasdale and Bryan Jennett at the University of Glasgow and published in Lancet [7]. Their Glasgow Coma Scale (GCS) is a widely used, rapid and reliable clinical tool for assessing the severity of a TBI. The three components include eye opening (1 to 4 points), motor response (1 to 6 points), and verbal response (1 to 5 points). The GCS score is used universally to grade the severity of a TBI. The TBI severity category correlates with the following GCS scores, 13–15 as mild, 9–12 as moderate, and, 3–8 as severe.

### 1.3. Criteria for Neuroimaging

It is widely accepted that for a moderate or severe closed head injury (GCS 3–12), the most appropriate initial neuroimaging study is a noncontrast computed tomography (CT) scan of the head [8,9,10,11,12]. A noncontrast CT scan can reveal critical life-threatening injuries such as an expanding epidural hematoma with impending herniation, which requires emergency neurosurgical evacuation. Noncontrast CT scans can also evaluate for many other types of injuries that direct management including fracture, intracranial hemorrhage, contusion, and associated mass effect.

Approximately 80% of the patients seen in the Emergency Department in the United States with a head injury are graded as a mild TBI (GCS 13–15). Clinical decision making tools to determine if a mild TBI patient needs a noncontrast head CT as part of their initial work-up include the National Emergency X-Ray Utilization Study (NEXUS)-II [13] clinical criteria, the Canadian CT Head Rules (CCHR) [14], the New Orleans Criteria (NOC) [15], and the Pediatric Emergency Care Applied Research Network (PECARN) for guidance of pediatric head injury assessment [16]. If patients with mild TBI are discharged, the patient must be alert and oriented enough to understand discharge instructions and must have a companion to stay with them for the next 24 h [17]. 

### 1.4. Primary Traumatic Brain Injury vs. Secondary Traumatic Brain Injury

According to the American College of Surgeons, a primary TBI is the brain damage that “occurs at the time of impact and produces its clinical effect almost immediately and is refractory to most treatment”. Some primary TBIs are due to penetrating injuries where an object traverses through the skull directly disrupting brain structures where management includes antibiotics and possible neurosurgical intervention [17]. Other TBIs are due to closed head injuries (CHI) where the damage is due to internal forces including linear or rotational acceleration/deceleration, stretching, or compression type forces. The damaging forces experienced during a CHI may result in contusion, damage to blood vessels and axonal shearing. A secondary TBI is the brain damage that “occurs at some time after the primary impact and is largely preventable and treatable”. Secondary TBIs include cerebral edema, altered cerebral blood flow, impaired metabolism, and free radical formation. According to the American College of Surgeons, for patients with suspected TBI, the primary goal of treatment is to prevent the secondary TBI [17].

## 2. Conventional Diagnostic Imaging Techniques in Traumatic Brain Injury

Traumatic brain injury can be extremely heterogeneous, ranging in mechanism, severity and location with primary and secondary TBI patterns. Conventional imaging techniques can classify the structural injury patterns in the brain. Traumatic brain injury can be hemorrhagic or nonhemorrhagic. Locations of intracranial hemorrhage can include the epidural space, subdural space, subarachnoid space, intraparenchymal, or intraventricular space.

**Figure 1:**
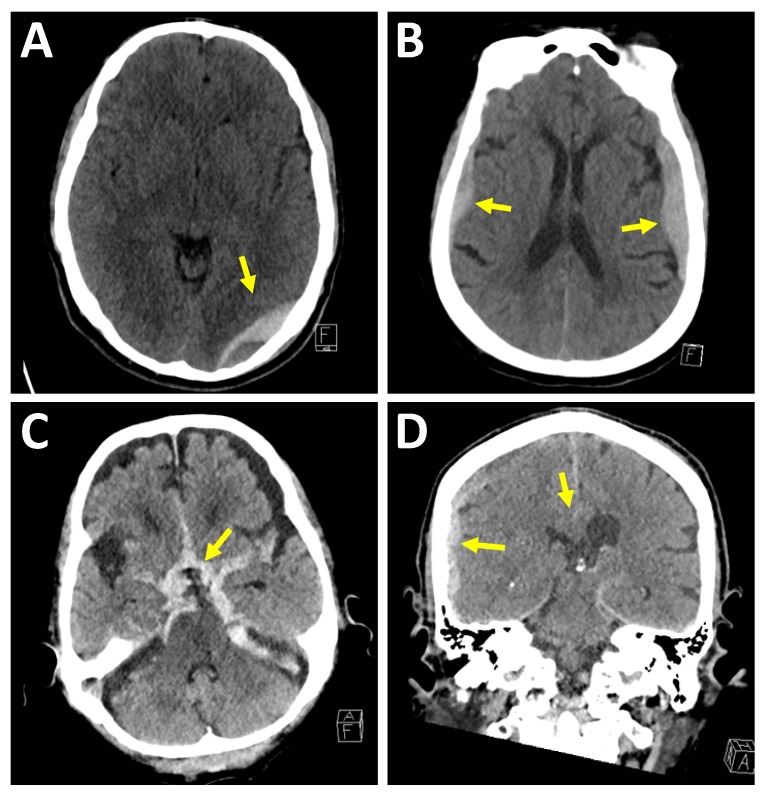
Extra-axial hemorrhage patterns. (**A**) Epidural hematoma. Thirty-three-year-old man was brought in by ambulance after a motor vehicle accident. Axial image demonstrates a biconvex shaped extra-axial fluid collection superficial to the left occipital lobe with hyperdense and hypodense components. Adjacent left temporal bone fracture not seen on this axial image. (**B**) Bilateral subdural hematomas. Seventy-three-year-old woman presented after a fall out of bed with dizziness. Axial image demonstrates bilateral crescentic shaped fluid collections, which were found to cross the coronal sutures. (**C**) Massive subarachnoid hemorrhage. Ninety-seven-year-old man on anticoagulation fell while walking. Axial image demonstrates a subarachnoid hemorrhage within the basal cisterns. Patient expired five days later due to cardiorespiratory failure. (**D**) Subdural hematoma with developing subfalcine herniation. Sixty-nine-year-old man on anticoagulation fell and struck his head. Coronal image demonstrates subdural hematoma along the right cerebral convexity with mass effect on adjacent lateral ventricle, midline shift, and subfalcine herniation.

In an epidural hematoma, the hematoma is located between the dura mater and the calvarium. Clinically, epidural hematomas can be associated with a lucid interval followed by clinical deterioration once a critical level of intracranial pressure (ICP) is reached [18]. Epidural hematomas are typically biconvex shaped, commonly associated with skull fractures and commonly arise from arterial bleeding, such as the middle meningeal artery in children. Since the middle meningeal artery enters the skull through the foramen spinosum at the floor of the middle cranial fossa, the neuroradiologist must look carefully at the floor of the middle cranial fossa on the coronal images for an epidural hematoma. Epidural hematomas can also be caused by venous injuries such as at the dural venous sinuses. On rare circumstances, an epidural hematoma can decompress outward through a skull fracture into the scalp. Epidural hematomas are most commonly encountered in the acute phase, see Figure 1.

In a subdural hematoma, the hematoma is located between the dura mater and the arachnoid membrane; therefore, subdural hematomas can travel across calvarial sutures and along the falx cerebri and tentorium cerebelli. Subdural hematomas are commonly caused by shearing of bridging veins, which can be due to the mobile, rotating brain with respect to the fixed dura mater [19]. Subdural hematomas generally involve a higher degree of underlying brain injury as compared to epidural hematomas. Underlying cerebral edema, mass effect, or midline shift may contribute to the feared complication of elevated ICP and cerebral herniation. Also, in contrast to epidural hematomas, which typically present in the acute phase, subdural hematomas commonly present at varying ages of hemorrhage, see Figure 1.

In subarachnoid hemorrhages, the hemorrhage is located within the subarachnoid space and can be seen in the sulci between the cortical gyri, the fissures between the cerebellar folia and the cisterns such as the basal cisterns or the perimesencephalic cistern. Once hemorrhage enters the subarachnoid space, it can undergo a variable amount of dilution by the cerebrospinal fluid. In addition to trauma, an important and common cause of subarachnoid hemorrhage is aneurysmal hemorrhage [20], see Figure 1.

**Figure 2:**
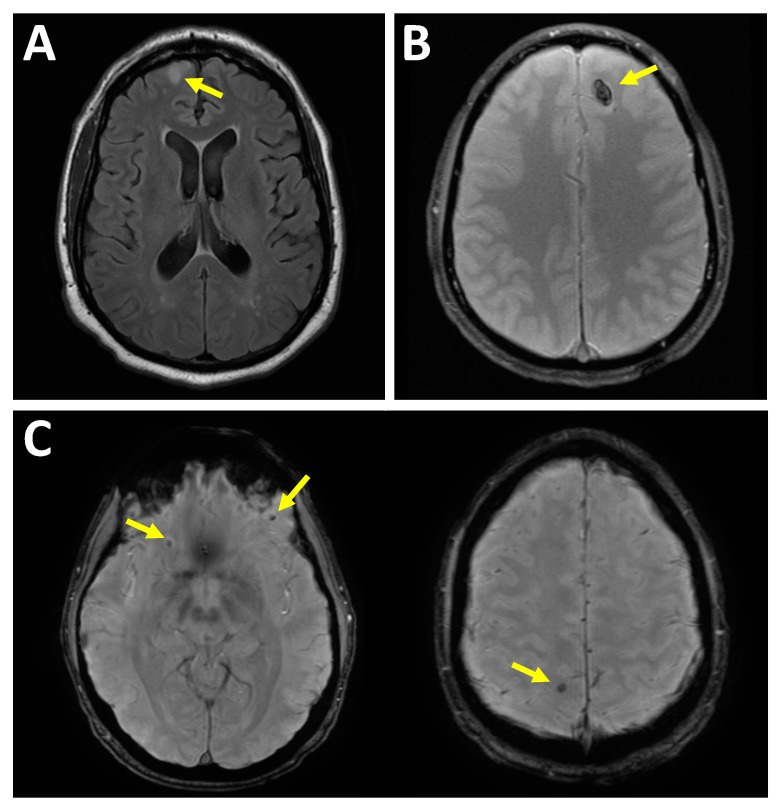
Intra-axial injury patterns. (**A**) Nonhemorrhagic brain surface contusion. Sixty-six-year-old male fell and hit head on concrete with subsequent loss of consciousness. Axial T2-weighted fluid-attenuated inversion recovery (FLAIR) image demonstrates focal increased signal within the right frontal lobe cortex. (**B**) Hemorrhagic brain contusion. Twenty-four-year-old male who fell and hit his head after playing basketball with subsequent loss of consciousness. Axial T2*-weighted gradient echo (GRE) image demonstrates focal region of hypointense signal in the left front lobe. (**C**) Diffuse axonal injury. Forty-year-old male after falling from multiple flights of stairs. Two axial T2* susceptibility-weighted imaging (SWI) images demonstrate multiple foci of hypointensity consistent with punctate hemorrhage at the gray-white junctions. This is a consequence of shearing forces.

In an intraparenchymal hematoma, the hemorrhage is within the brain tissue itself. Intraparenchymal hemorrhages are commonly due to contusions and axonal injuries. The mechanisms of injury in diffuse axonal injury (DAI) are likely more complex than only primary mechanical axotomy in the setting of trauma [21,22,23]. Diffuse axonal injury is a shearing-type injury to axons typically from a significant rotational acceleration/deceleration force [24]. On MRI, DAI injuries can be seen as hyperintense signal on a T2-weighted sequence and, if associated with hemorrhage then hypointense signal on T2*-weighted sequence, at the corticomedullary junction, corpus callosum, internal capsule, brainstem, and cerebellum [25]; see Figure 2.

A noncontrast CT scan is the most appropriate initial neuroimaging examination for moderate or severe TBI. There are, however, several limitations to noncontrast CT scans of the head, including the underestimation of parenchymal contusions in the early post-trauma period, the limitation in detecting diffuse axonal injury and the limitation of detecting secondary ischemic changes related to cerebral edema and intracranial hypertension [26,27,28,29].

## 3. Advanced Diagnostic Imaging Techniques in Traumatic Brain Injury

Advanced diagnostic imaging techniques go beyond routine CT and MRI protocols and may provide important prognostic information, improve treatment strategies, and improve clinical outcomes.

### 3.1. Perfusion Imaging

Perfusion imaging is an advanced neuroimaging technique that provides important information including cerebral blood flow, blood volume, and blood transit time. While perfusion imaging has most commonly been used in the setting of stroke [30,31,32,33], it also currently being researched in brain death [34] and TBI [35]. In this section, we will discuss clinical considerations as it pertains to TBI, perfusion imaging techniques and results from important perfusion imaging studies. 

#### 3.1.1. Clinical Considerations

The primary goal for the treatment of patients with TBI is to prevent secondary injury [17,36,37]. Nearly half of TBI-related deaths after admission are thought to be due to secondary ischemic changes related to cerebral edema and intracranial hypertension [29].

Cerebral autoregulation is the process by which the cerebral vasculature either vasodilates or vasoconstricts to maintain appropriate levels of cerebral blood flow over a range of blood pressures. In a healthy patient, cerebral blood flow can be autoregulated over mean blood pressure ranging from 50 mmHg to 150 mmHg [17]. In the setting of TBI, the ability of the cerebral vasculature to perform autoregulation is impaired; therefore, optimal management strategies include maintaining normal blood pressure, avoiding systemic hypotension, and avoiding low arterial oxygen saturation [17]. Additionally, optimal clinical care includes maintaining normal ICP.

**Figure 3:**
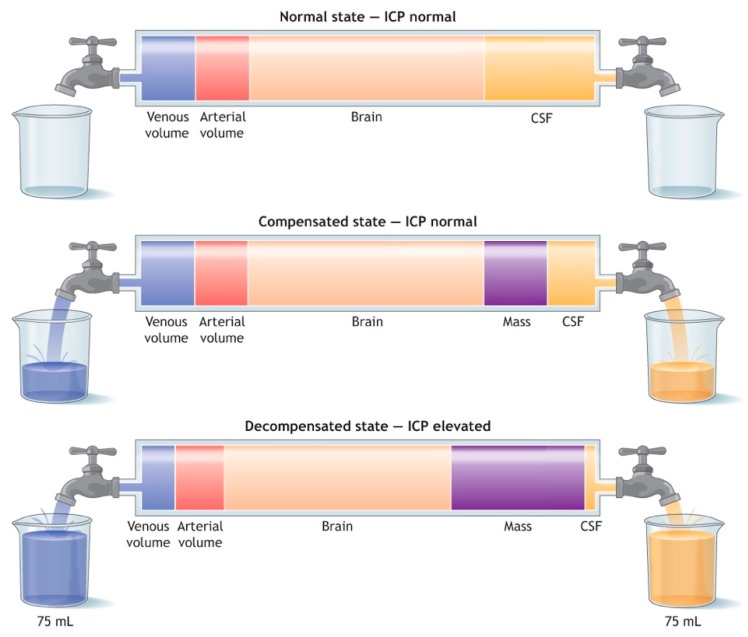
This figure illustrates the relationship between the volume of the intracranial mass (e.g., epidural hematoma), the volume of the intracranial venous blood, the volume of intracranial arterial blood, the volume of brain, the volume of cerebrospinal fluid (CSF), and the intracranial pressure (ICP). Initially, as the mass enlarges, venous blood and CSF are expelled out of the intracranial space and the ICP remains normal, which is referred to as the compensated state. If the extra-axial hematoma continues to increase, a decompensated state will be reached and the ICP will elevate with increasing mass volume. Reprint from the Advanced Trauma Life Support Tenth Edition Head Trauma lecture with permission from the American College of Surgeons [17].

**Figure 4:**
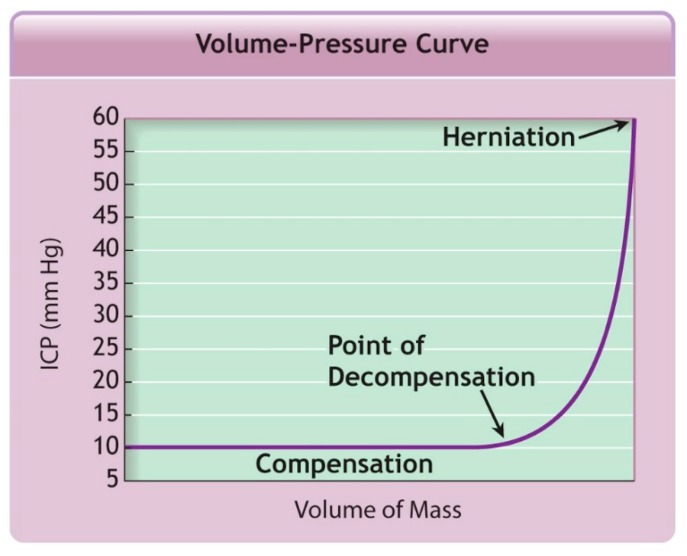
This figure illustrates the relationship between the volume of the mass (e.g., an epidural hematoma) and the ICP. Initially, as the mass enlarges, the ICP remains normal. This is referred to as the compensated state. If the volume of mass hematoma continues to increase beyond the point of decompensation, the ICP will rapidly elevate with increasing mass volume. This is referred to as the decompensated state and, in the absence of urgent intervention, will ultimately result in herniation. Reprint from the Advanced Trauma Life Support Tenth Edition Head Trauma lecture with permission from the American College of Surgeons [17].

The Monro–Kellie doctrine, based on hemostatic intracranial volume regulation, describes the relationship between the volume of an intracranial hematoma and ICP. Under normal circumstances, the rigid skull is filled with the brain, intravascular blood and cerebrospinal fluid. In the setting of an expanding intracranial hematoma, the ICP changes over two phases, see Figure 3 and Figure 4. During the first phase, some of the intravascular blood is pushed out of the skull, some of the cerebrospinal fluid is absorbed and the ICP remains normal. During the second phase, this compensatory mechanism is exhausted and there is an exponential rise in the ICP for even a small additional increase in the volume of the intracranial hematoma. The cerebral perfusion pressure (CPP) is equal to the mean arterial pressure (MAP) minus the ICP. Thus, for a constant MAP, a rapid rise in ICP can significantly drop the CPP and cerebral blood flow (CBF), which in turn will compromise oxygen and metabolite delivery [17].

As previously discussed, a noncontrast CT scan is limited in the assessment of early ischemic changes. Imaging of these secondary ischemia changes may help to save the potentially salvageable tissue known as “traumatic penumbra” and improve clinical outcomes following TBI [38,39,40]. 

#### 3.1.2. Perfusion Imaging Techniques

Perfusion imaging is most commonly performed with CT and MRI. In perfusion CT (PCT), an intravenous nondiffusible (i.e., remains in the vasculature) contrast is administered and serial CT images are acquired to calculate perfusion parameters. In MRI, perfusion can be performed via two methods. First, a nondiffusible (i.e., remains in the vasculature) gadolinium-based contrast agent can be administered during an MRI examination. This is called bolus perfusion MRI and an example is called Dynamic Susceptibility Weighted Contrast (DSC) [41]. Second, a noncontrast technique called arterial spin labeling (ASL) can be performed by magnetically labeling endogenous blood water as a diffusible flow tracer [32,41,42,43,44,45].

A key principle in perfusion imaging is known as the central volume principle, which provides the relationship between the compartment volume, blood flow through the compartment, and the mean transit time through the compartment [33]. Important output measures available through perfusion imaging include cerebral blood volume (CBV), (CBF), mean transit time (MTT), and time of maximum concentration (Tmax) [46,47,48,49,50]. The units of CBV are mL of blood/100 g of brain tissue. The mean transit time is the average time for blood to flow from the arterial input to the venous drainage and has units in seconds. The units of CBF are mL of blood/100 g of brain/minute. The time of maximum concentration is calculated from the time to peak of the residue function [51]. If a more thorough review of perfusion imaging techniques is desired, we would like to refer readers to one of our prior manuscripts that discusses perfusion imaging in greater depth [35].

#### 3.1.3. Results of Important Studies of Perfusion Imaging of Traumatic Brain Injury

**Figure 5:**
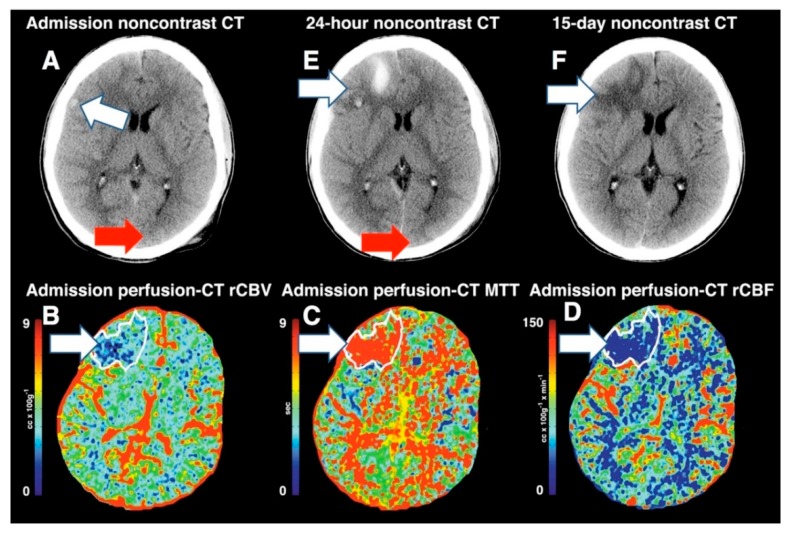
Noncontrast and perfusion computed tomography (PCT) images from a patient with severe traumatic brain injury (TBI). (**A**) Noncontrast CT at admission revealed a small hemorrhagic contusion in the right frontal lobe (arrow). Admission PCT images demonstrates a large territory of decreased regional cerebral blood volume (rCBV) (**B**), increased mean transit time (MTT) (**C**) and decreased regional cerebral blood flow (rCBF) (**D**). Follow-up noncontrast CT at 24 h (**E**) demonstrates increased areas of hemorrhagic contusion in the right frontal lobe where the perfusion abnormality was seen. Follow-up noncontrast CT at 15-days (**F**) demonstrates evolving hemorrhagic contusion and encephalomalacia in the right frontal lobe, which corresponds to the same distribution that is seen on the perfusion-CT on admission. Reprinted with permission from [35].

**Figure 6:**
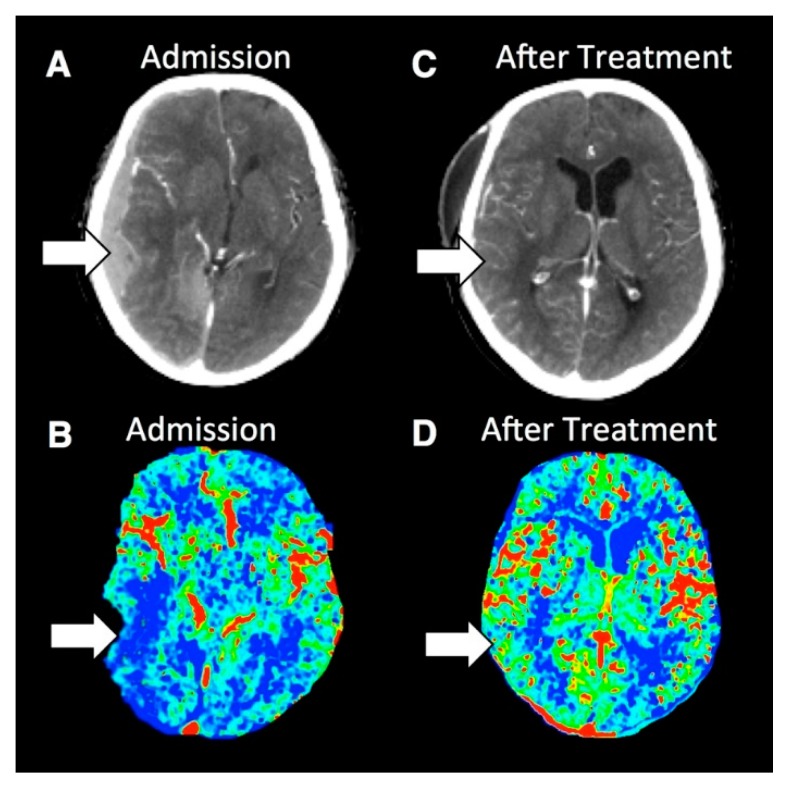
Contrast-enhanced and PCT images from a patient with severe TBI. (**A**) Contrast-enhanced CT imaging at admission demonstrates a right-sided subdural hematoma causing mass effect on the underlying brain and midline shift. (**B**) rCBF PCT imaging at admission demonstrates decreased rCBF in the right temporal lobe. (**C**) Contrast-enhanced CT image after surgical evacuation of the hematoma demonstrates resolution of the right-sided subdural hematoma, mass effect and midline shift. (**D**) rCBF PCT imaging after surgical evacuation of the right-sided hematoma demonstrates normalization of the rCBF in the right temporal lobe. Reprinted with permission from [35].

**Figure 7:**
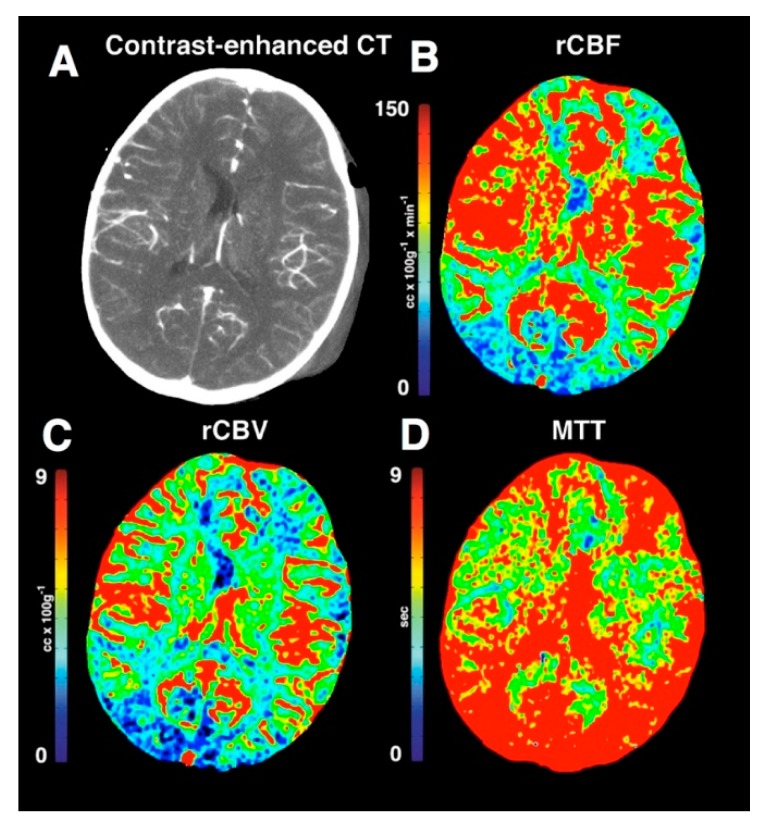
Contrast-enhanced and PCT images of a case of TBI with intracranial hypertension. The contrast enhanced CT (**A**) demonstrated left-sided scalp hematoma. The rCBF (**B**) and rCBV (**C**) trended toward lower values especially in the occipital lobes. The MTT (**D**) demonstrated significantly higher values, reflecting altered cerebral autoregulation after TBI. Reprinted with permission from [35].

Just as TBI is a heterogenous pathology, so too are the patterns of perfusion abnormalities seen in TBI. Multiple studies have documented perfusion defects seen in TBI patients in a variety of sites throughout the brain [36,52,53,54,55,56,57]. Abnormal perfusion has been identified in the context of cerebral edema, extra-axial collections, and intracranial hypertension [58,59], see Figure 5, Figure 6 and Figure 7. One study has suggested that PCT may be able to distinguish whether an area of hypodensity on noncontrast CT is necrotic or viable, something that is not possible on noncontrast CT alone [60]. Still further, PCT provides insight into cerebral autoregulation, which can be used to help guide therapy [60]. 

In cases of normal noncontrast CT head examinations, PCT with reductions in CBF and CBV was associated with worse clinical outcomes [61,62]. In severe TBI, PCT showed additional information in 60% of patients and altered management in 10% of patients [63,64]. 

#### 3.1.4. Limitations of Perfusion Imaging in Traumatic Brain Injury

There are several drawbacks to perfusion imaging in the setting of TBI. First, PCT is associated with radiation dose. The ordering physician and diagnostic radiologist should weigh the risks of ionizing radiation versus the benefit of obtaining a diagnostic scan. The diagnostic radiology department must make every effort for to achieve radiation reduction strategies to mitigate risks associated with ionizing radiation. With respect to radiation dose, the goal of every radiology practice should be to keep the dose as low as reasonably achievable (ALARA) and adhere to radiology practice guidelines [65]. Second, some perfusion examinations administer contrast material, which carry the risks of contrast reaction or renal impairment [66]. Third, both PCT and MRI perfusion imaging examinations take extra time, which may be impractical in the setting of trauma. Finally, some patients have known contraindications to MRI or are not cleared for MRI in the setting of trauma. The authors of this paper feel that perfusion imaging has significant potential in improving neuroimaging diagnosis of acute TBI; however, further research needs to be performed.

### 3.2. Diffusion Tensor Imaging

Diffusion tensor imaging is an advanced neuroimaging technique that provides important information on the connections within the brain. Diffusion tensor imaging provides important information on the macroscopic, structural connectivity of the brain (as opposed to other techniques that provide information about functional connectivity). In this section, we will discuss clinical considerations, DTI imaging techniques and results from important studies. 

#### 3.2.1. Clinical Considerations

As previously discussed, the human brain is comprised of 100 billion neurons that communicate with each other via dendrites and axons [6]. A microscopic view of the superficial gray matter reveals radially-oriented layers of neuron cell bodies, which intercommunicate via a complex, branching network of dendrites. A part of the neuron called the axon dives deeper into the white matter of the brain. Axons travel in clusters called tracts to nearby or remote regions of the brain. The axons are wrapped in myelin, which serves to insulate the traveling electrical signal and connect functionally specialized, yet segregated, regions of the brain. As the white matter tract approaches its destination, it fans outwards into the cortex and terminates in one or more synaptic contacts. In the setting of TBI, such axons can be exposed to a wide variety of forces including compression, tension, shear, bending and torsion forces. Therefore, there is strong rationale for performing DTI to image the neuroanatomic connectome and assess for axonal injury.

#### 3.2.2. Diffusion Tensor Imaging Techniques

**Figure 8:**
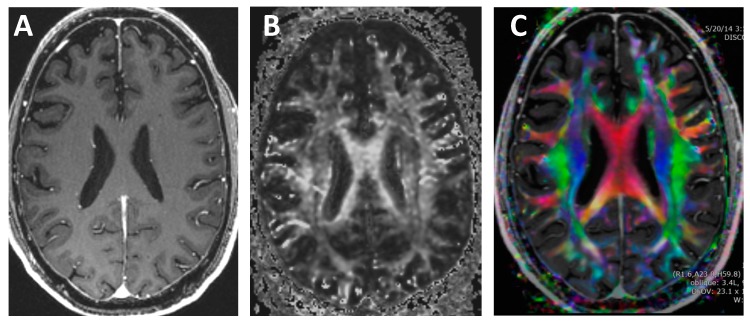
T1-weighted and diffusion tensor imaging (DTI). (**A**) Contrast-enhanced T1-weighted image. Note that the white matter is all the exact same grayscale and the direction of each white matter tract cannot be discerned. (**B**) Grayscale DTI anisotropy map for one diffusion-sensitizing gradient. Note that there are varying grayscales within the white matter, which correspond to the amount of diffusion signal for the particular directional diffusion-sensitizing gradient applied during the acquisition. (**C**) Color DTI anisotropy map overlaid onto a T1 post-contrast image. The Color DTI anisotropy is based on the composite of multiple diffusion-sensitizing gradient images. Note that there are multiple colors within the white matter map with red indicating transverse direction, blue indicating superior–inferior direction and green indicating anterior-posterior direction.

Conventional MRI imaging techniques do not provide information on the direction of the white matter tracts. In contrast, DTI quantifies both the asymmetry and the amount of water diffusion and displays this information on color-coded maps [6,67,68,69,70,71,72,73,74,75], see Figure 8. The concept of diffusion can be thought of as an ink drop falling onto a piece of wood. The rate of growth will be fastest in the direction along with the grains of the wood. Likewise, water diffusion is axons is directionally dependent. 

The DTI sequence is a spin-echo diffusion-weighted pulse sequence with diffusion weighting in multiple different spatial directions using diffusion-sensitizing gradients. A typical DTI protocol will have a typical slice thickness of 2 mm and matrix of 128 × 128 [76,77,78]. For each diffusion-sensitizing gradient, the 4D dataset includes x, y, z spatial locations with a diffusion constant proportional to the magnitude of diffusion. A minimum of six diffusion-sensitizing gradients are used; however, modern protocols typically include 30 directions [79]. 

**Figure 9:**
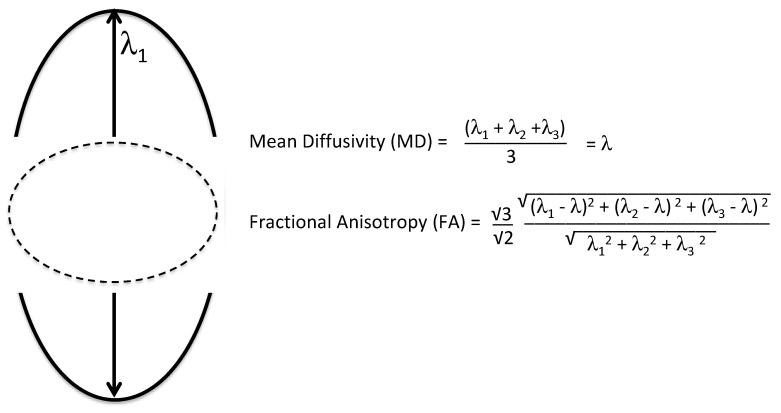
Illustration of 3D ellipsoid and formulas for mean diffusivity (MD) and fractional anisotropy (FA). The 3D ellipsoid is characterized with three eigenvectors that define the axes and with three associated eigenvalues (λ) that define the lengths.

Two important DTI metrics include fraction anisotropy (FA), which is a measure of the asymmetry of diffusion and mean diffusivity, which is a measure of the magnitude of diffusion [80]. Formulas for FA and mean diffusivity (MD) are shown in Figure 9. Normal white matter is highly anisotropic and has high FA and low MD; however, during traumatic axonal injury, white matter FA and MD can be altered. 

**Figure 10:**
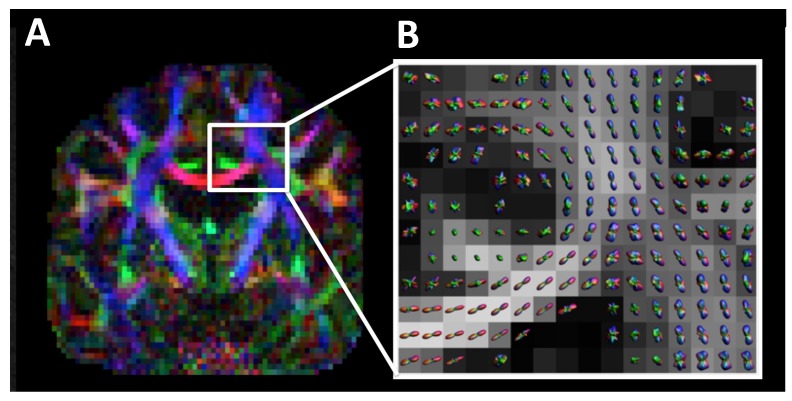
Example of Q-ball imaging (QBI). (**A**) QBI type DTI with color anisotropy map full field of view. (**B**) Zoomed in region from the white rectangle in (A) with probability distributions in each voxel superimposed on a grayscale FA map. QBI acquisition parameters: 112 × 112 matrix; 22.4 × 22.4 cm field-of-view; 70 axial slices of 2 mm thickness; 6 b.0 images; 60 gradient directions at b.2500 s/mm^2^; SENSE acceleration factor 2; TE/TR.107 ms/10.3 s; and, acquisition time 11 m 20 s.

One of the technical limitations that many researchers are attempting to overcome is the ability to assess intravoxel fiber tract crossing [76,81,82,83,84]. Some of the newer DTI techniques including diffusion kurtosis imaging (DKI), Q-ball, neurite orientation dispersion and density imaging (NODDI), and Diffusion Spectrum Imaging (DSI) attempt to overcome these limitations [85,86,87,88,89,90,91,92,93,94,95,96], see Figure 10. If a more thorough review of DTI techniques is desired, we would like to refer readers to one of our prior manuscripts that discusses DTI in greater depth [76].

#### 3.2.3. Results of Important Studies of Diffusion Tensor Imaging of Traumatic Brain Injury

Numerous advanced neuroimaging studies on traumatic brain injury have focused on diffusion tensor imaging. A common finding across these studies is lower FA and higher MD in the TBI population as compared to the control group [97,98,99,100,101,102,103,104,105,106,107,108,109,110,111,112,113,114]. As an example, one study performed on National Football League (NFL) players showed decreased FA in the bilateral frontal and parietal lobes, the corpus callosum, and the left temporal lobes in the cognitively impaired football player group compared to controls [114]. There is significant heterogeneity of what regions within the brain show decreased FA, and this may be related to the heterogeneous nature of TBI. 

A recent systematic review and meta-analysis by Wallace and colleagues of 44 studies of DTI changes in mild, moderate and severe TBI showed a variety of brain regions involved [115]. In the analysis of the mild TBI subgroup, 88% of the all of the regions examined had significantly lower FA values in the mild TBI subgroup as compared to the controls. In the analysis of the moderate–severe TBI subgroup, 92% of all of the regions were found to have lower FA values compared with the controls. When Wallace and colleagues looked at MD, 95% and 100% of all of the regions examined were higher in MD in the TBI group compared to the controls for mild TBI and moderate–severe TBI, respectively. It should be noted that many of these studies were performed in the subacute or chronic post-TBI time periods. In the setting of acute moderate to severe TBI, the most appropriate initial study is a noncontrast CT scan. 

#### 3.2.4. Limitations of Diffusion Tensor Imaging in Traumatic Brain Injury

Population-based research studies have revealed that DTI is sensitive for TBI; however, this is only true at the group level. At this time, there is insufficient evidence to suggest that DTI can be used to diagnose TBI on the individual patient level [76,116,117,118]. In addition, the finding of decreased FA seen in the TBI populations lacks specificity. In fact, a variety of neurological conditions, especially those that affect white matter, can result in decreased FA [119]. 

## 4. Future Technologies

A key challenge in both optimally managing and researching TBI is the significant heterogeneity in TBI. There is heterogeneity found in the patient populations, comorbidities, mechanism of injury, site of injury, timing of injury and subsequent care received, imaging protocols, treatment, and follow-up. This heterogeneity of TBI poses a significant challenge in identifying the optimum treatment at the individual level [120]. In fact, some neuroprotective strategies which have positive outcomes in animal research have failed to translate to improved clinical outcomes in clinical TBI trials [121,122,123,124]. A possible explanation for this is the fact that TBI in clinical settings is more varied as compared to animal research [121,125,126]. One such technique that holds promise in improving research and helping patient care may be achieved through machine learning.

### Machine Learning in Traumatic Brain Injury

Machine learning is a form of artificial intelligence, which has shown promise in solving many problems in computer vision, robotics and language processing [127]. In the annual ImageNet Challenge, teams compete to develop algorithms to accurately classify millions of images into categories, such as dogs, cats, etc. The year 2012 represented a milestone for deep learning, since it was the first year that the neural network type of machine learning won the competition [128]. A neural network has won the competition every year since, demonstrating that such models may better capture the architecture of images. Deep learning neural network algorithms are already being applied to a variety of radiology processes, such as bone age radiographs, cerebral aneurysm detection on magnetic resonance angiography (MRA) and reducing volume of gadolinium administration [129,130,131]. 

Machine learning algorithms are being applied to traumatic brain injury as well. In one study, a machine learning algorithm was used in attempt to identify patients in whom a head CT can be avoided [132]. In another study, a machine learning algorithm was used to detect intracranial hemorrhage [133]. In other studies, machine learning algorithms were used to evaluate white matter tracks [134,135]. Outcomes of TBI were predicted using deep-learning algorithms using DTI [136]. A combination of support vector machines (SVMs) and deep learning algorithms were combined to identify hyperdense lesions associated with TBI using CT [137]. For example, support vector machines identified that cortical thickness was decreased compared to age-matched controls in military personal who had experienced TBIs [138]. Many other machine learning studies are being published in TBI in attempt to help radiologists in diagnosis of TBI and clinicians in management of TBI.

Steps forward for neuroimagers include the creation of a normal age-stratified imaging database. With a normative database, machine learning could be more effectively performed yielding a more accurate diagnosis through etiological, symptom-based or prognostic classifications of TBI [139,140,141,142]. At the TBI workshop on 23 May 2014 in Montreal, Canada, the American Society of Neuroradiology (ASNR), American College of Radiology (ACR), Head Injury Institute (HII), and American Society for Functional Neuroradiology (ASFNR), TBI experts discussed the formation of a consensus of the ideal database, the normal control subject, and standardizing clinical and research neuroimaging protocols [143].

## 5. Conclusions

Traumatic brain injury is a major public health challenge and unfortunately affects millions worldwide. In this review article, we discussed neuroanatomy, normal cerebral autoregulation, types of traumatic brain injury and intracranial hemorrhage, advanced imaging techniques including perfusion, diffusion tensor imaging, and future techniques including machine learning.
